# Supplementary material for: Comparison of two sentinel species Gammarus fossarum and Dreissena polymorpha for monitoring fecal viral contamination of continental waters
Source: Microbiol Spectr. 2026 Mar 17;14(4):e01262-25. doi: 10.1128/spectrum.01262-25 (PMC13055396; doi:10.1128/spectrum.01262-25)
Supplement: Supplemental figures and table — Figures S1 to S4 and Table S1. [file spectrum.01262-25-s0001.pdf]

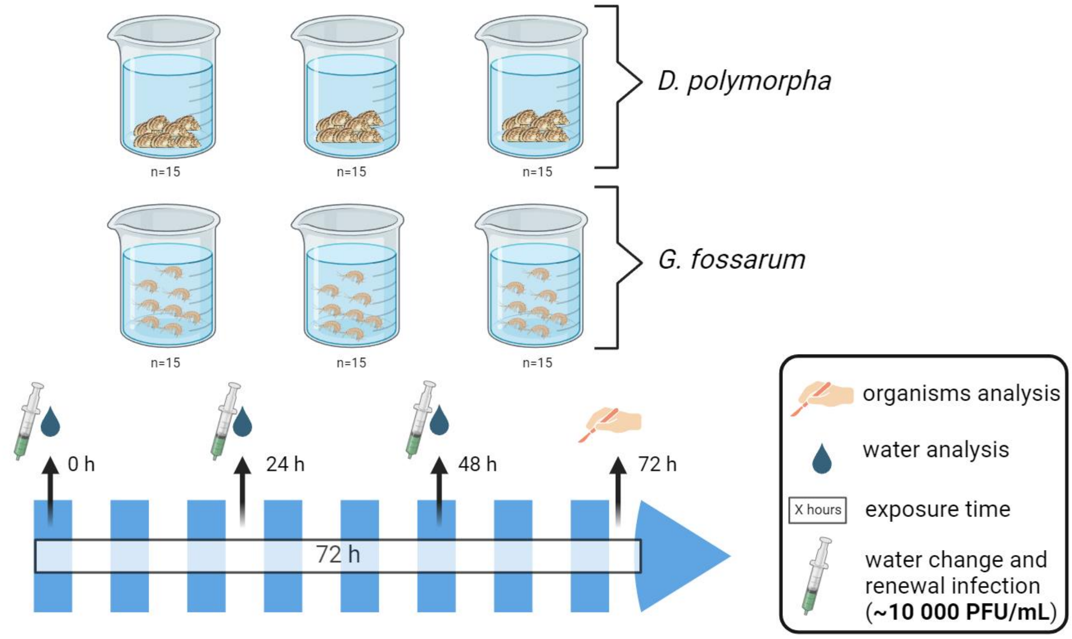

Figure S1: Details of experimental design and timeline for laboratory exposure.

Experimental design

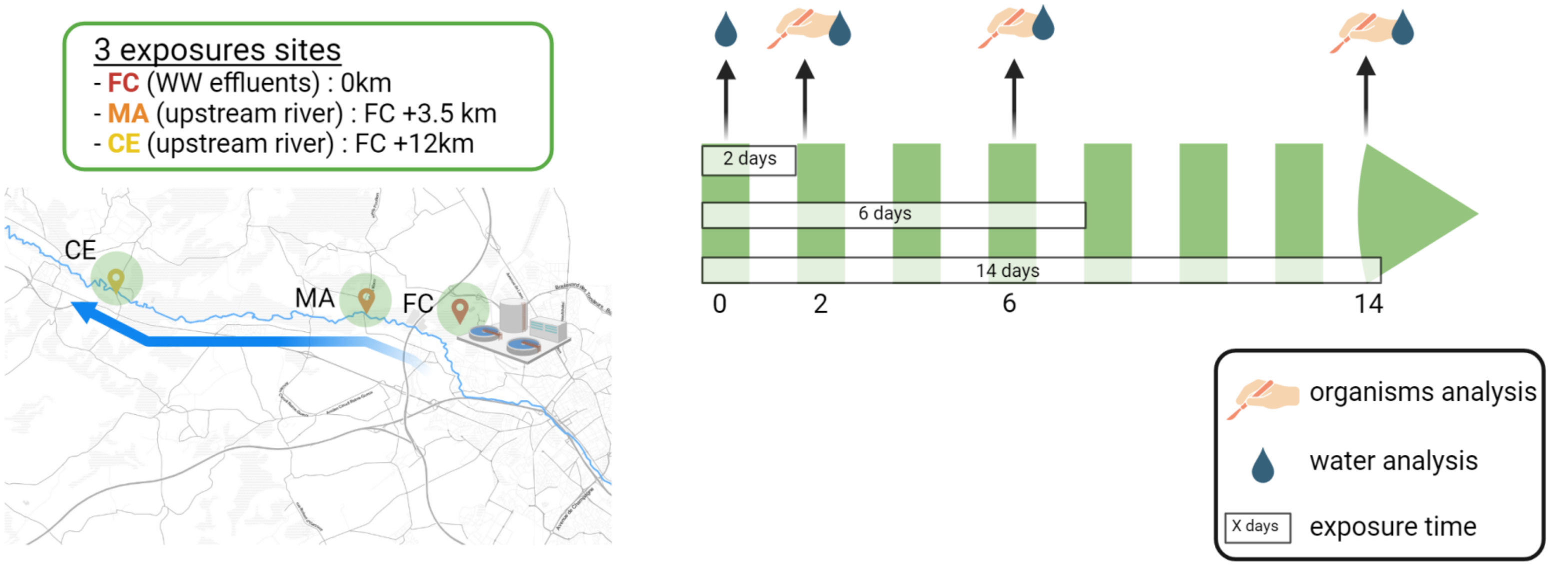

Figure S2: **Details of experimental design and timeline for *in situ* exposures.**

		FC	MA	CE
**Temperature** (°C)	Mean	18.89	14.88	14.48
	SD	1.21	1.15	0.88
	n	336	336	336

<u>Table S1</u>: Average values of abiotic parameters measured during 14 days of exposure following exposure sites. Exposure sites are WWTP effluent (FC), 3.5km (MA) and 12km (CE) on the Vesle river, downstream of the WWTP. Significant differences between sites are indicated in the "Sign." line ; if two sites have the same letter, they are not significantly different according to the Kruskal-Wallis test (p-value > 0.05).

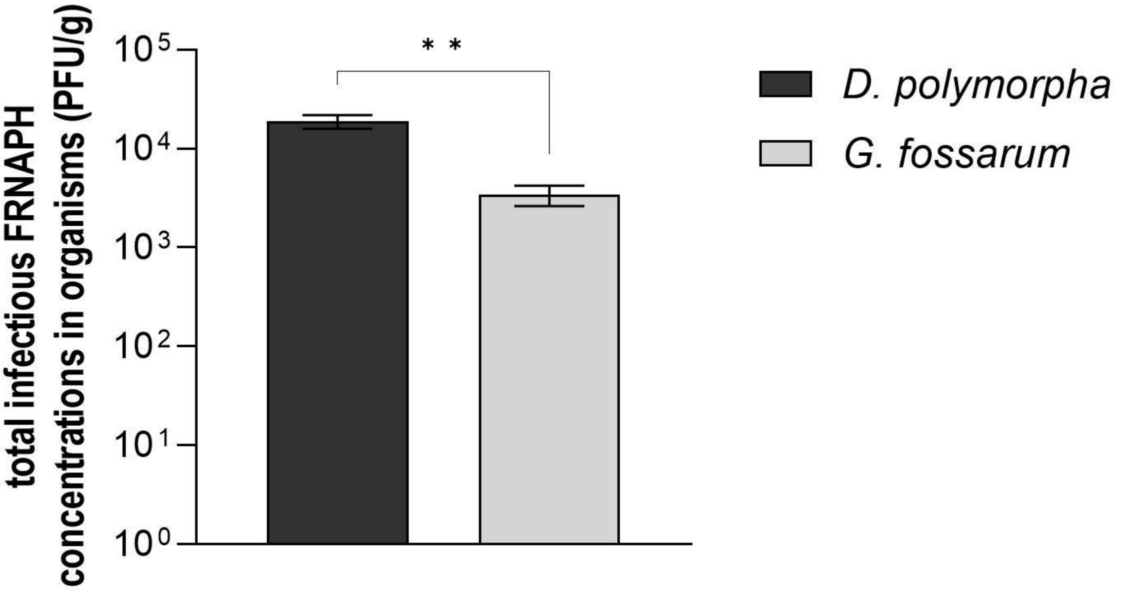

<u>Figure S3</u>: **Average concentration of total infectious FRNAPH (PFU/g) measured in the two species exposed in the laboratory to the same conditions.** Error bars represent the mean with standard deviation (SD). Exposure condition labeled with asterisks are significantly different (** <0.01) according to the Student test.

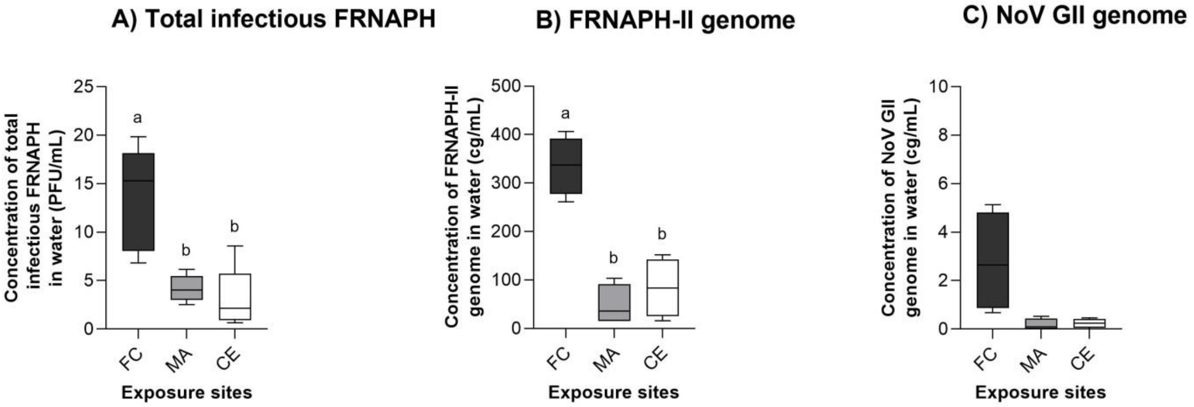

<u>Figure S4</u>: Viral target concentrations measured in water samples (n=5) following exposure sites : A) total infectious FRNAPH (PFU/mL) and B) FRNAPH-II genome (cg/mL) and C) NoV GII genome (cg/mL). Error bars represent the mean with standard deviation (SD). Exposure sites are WWTP effluent (FC), 3.5km (MA) and 12km (CE) on the Vesle river, downstream of the WWTP. Sites with the same letter are not significantly different according to the Kruskal-Wallis test (p-value > 0.05).
